# Group B streptococcal colonization in elderly women

**DOI:** 10.1186/s12879-021-06102-x

**Published:** 2021-05-03

**Authors:** Rossella Baldan, Sara Droz, Carlo Casanova, Laura Knabben, Dorothy J. Huang, Christine Brülisauer, André B. Kind, Elke Krause, Stefanie Mauerer, Barbara Spellerberg, Parham Sendi

**Affiliations:** 1 Institute for Infectious Diseases, University of Bern, Friedbühlstrasse 51, 3010 Bern, Switzerland; 2 Department of Gynecology and Obstetrics, Inselspital, Bern University Hospital and University of Bern, Bern, Switzerland; 3 Outpatient Department & Colposcopy Unit, University Women’s Hospital Basel, Basel, Switzerland; 4 Institute of Medical Microbiology and Hygiene, University Hospital Ulm, Ulm, Germany; 5 Division of Infectious Diseases & Hospital Hygiene, University Hospital Basel and University of Basel, Basel, Switzerland

**Keywords:** Group B Streptococcus, *Streptococcus agalactiae*, Colonization, Elderly women, Postmenopausal women

## Abstract

**Background:**

In non-pregnant adults, the incidence of invasive Group B *Streptococcus* (GBS) disease is continuously increasing. Elderly and immunocompromised persons are at increased risk of infection. GBS commonly colonizes the vaginal tract, though data on colonization in the elderly are scarce. It is unknown whether the prevalence of GBS colonization is increasing in parallel to the observed rise of invasive infection. We conducted a three-year (2017–2019) prospective observational cross-sectional study in two teaching hospitals in Switzerland to determine the rate of GBS vaginal colonization in women over 60 years and i) to compare the proportions of known risk factors associated with invasive GBS diseases in colonized versus non-colonized women and ii) to evaluate the presence of GBS clusters with specific phenotypic and genotypic patterns in this population.

**Methods:**

GBS screening was performed by using vaginal swabs collected during routine examination from women willing to participate in the study and to complete a questionnaire for risk factors. Isolates were characterized for antibiotic resistance profile, serotype and sequence type (ST).

**Results:**

The GBS positivity rate in the elderly was 17% (44/255 positive samples), and similar to the one previously reported in pregnant women (around 20%). We could not find any association between participants’ characteristics, previously published risk factors and GBS colonization. All strains were susceptible to penicillin, 22% (8/36) were not susceptible to erythromycin, 14% (5/36) were not susceptible to clindamycin and 8% (3/36) showed inducible clindamycin resistance. Both M and L phenotypes were each detected in one isolate. The most prevalent serotypes were III (33%, 12/36) and V (31%, 11/36). ST1 and ST19 accounted for 11% of isolates each (4/36); ST175 for 8% (3/36); and ST23, ST249 and ST297 for 6% each (2/36). Significantly higher rates of resistance to macrolides and clindamycin were associated with the ST1 genetic background of ST1.

**Conclusions:**

Our findings indicate a similar colonization rate for pregnant and elderly women.

**Trial registration:**

Current Controlled Trial ISRCTN15468519; 06/01/2017

**Supplementary Information:**

The online version contains supplementary material available at 10.1186/s12879-021-06102-x.

## Background

Group B Streptococcus (GBS, *Streptococcus agalactiae*) is a pathobiont frequently found in the normal microbiota of the gastrointestinal and vaginal tracts of women [1, 2]. GBS can cause life-threatening infections in neonates, with maternal colonization being the principal route of transmission. In non-pregnant adults, the incidence of infection is continuously increasing [3, 4]. Elderly and immunocompromised persons with underlying conditions, such as diabetes mellitus and cancer, are at increased risk of invasive GBS disease [4–6]. A Danish study showed that from 2005 to 2018, the incidence of invasive GBS in adults aged 65–74 years increased from 3.23 to 8.34 per 100,000, and in adults over 75 years from 6.85 to 16.01 per 100,000 [7]. Their finding aligns with data from Iceland, Finland, Norway, England and Wales, Canada, and other countries [8–12]. Skin and soft-tissue infection, primary bacteraemia and urinary tract infection are the most frequent clinical manifestations of invasive GBS disease in the elderly [13, 14]. Most studies have investigated the prevalence of GBS colonization in pregnant women, only a few focusing on non-pregnant adults [13, 15]. Vaginal colonization in pregnant women worldwide ranges between 5 and 30%–35%, with an average estimate of approximately 20% [14, 16]. In contrast, little is known about the GBS colonization rate in women older than 60 years of age. It is unknown whether the prevalence of GBS colonization is increasing in parallel to the observed rise of invasive infection. The site of GBS colonization could potentially be the source of invasive infection, underscoring the importance to investigating the colonization rate in this patient population.

We present here the results of a prospective observational cross-sectional study in which we aimed to determine the vaginal colonization rate in women over the age of 60 in two teaching hospitals in Switzerland. Secondary objectives of the study were to compare the proportions of known risk factors associated with invasive GBS diseases in colonized versus non-colonized women and to evaluate the presence of clusters with specific phenotypic and genotypic patterns in GBS strains isolated in our population.

## Methods

### Study design and participants’ data

Women presenting at the outpatient clinic of two centres (Bern and Basel University hospitals) for a routine vaginal examination between January 2017 and December 2019 were screened for eligibility. Participants to be included in the study had to be ≥60 years old and capable of reading and understanding the patient information sheet and giving voluntary written consent to participate in the study, in which a vaginal swab would be collected during their routine gynaecological examination and cultured for the presence of GBS. In addition, participants were asked to complete a short questionnaire to obtain data regarding ethnicity, current or prior medical conditions, menstrual history and sexual history (Supplementary Material). Patient consent, study information and questionnaires were available in four languages (German, French, Italian and English). The swab and the questionnaire were coded to protect participants’ identifiable data and privacy, according to the approved study protocol (trial registration no. ISRCTN15468519). The study was approved by the local ethical committee (Kantonale Ethikkommission-Bern: 2016–01669).

### GBS culture and characterization

GBS screening was carried out with the same methodology throughout the whole study period. Isolation of the strain from vaginal samples was performed by growth in an enrichment medium (Todd–Hewitt broth) followed by subculture on a selective GBS chromagar plate (StrepB, CHROMagarTM, Paris, France). Identified colonies were subjected to MALDI-TOF mass spectrometry.

GBS isolates were further characterized at the phenotypic and genotypic level to determine the antibiotic resistance profile, the capsular serotype and the clone sequence type (ST). The minimal inhibitory concentrations (MICs) for penicillin, clindamycin and erythromycin were determined by E-test (bioMérieux, Marcy l’Etoile, France) and interpreted according to CLSI guidelines [17]. Detection of the macrolide-lincosamide-streptogramin B (MLS_B_) resistance phenotype was assessed by double disk diffusion test [18, 19]. Capsular serotyping was performed by use of a rapid latex agglutination test and polymerase chain reaction analysis, as previously described [20, 21] Sequence type was determined by multilocus sequence typing as described elsewhere (https://pubmlst.org/sagalactiae/). One sample per patient was included in the analysis. In the case of multiple sampling from the same participant, only the swab obtained at the first visit was analysed.

### Data analysis

Questionnaire and microbiology data were recorded in an electronic database designed with REDCap software (Research Electronic Data CAPture). Significant associations between participants’ characteristics/risk factors, GBS carrier status, GBS antibiotic resistance profiles, serotypes and sequence types were investigated. In the case of missing answers per single question, the denominator for each analysis was adapted accordingly. GraphPad Prism 8.0 was used for statistical analysis. The association between age variables and a positive GBS result was investigated by using the Mann-Whitney test. Differences in the prevalence of risk factors between GBS-positive and GBS-negative groups were assessed by contingency tables and the chi-square test, or by Fisher’s exact probability test if the frequency was less than 5. For direct comparisons, distribution analyses were also performed by using the chi-square test, Fisher’s exact test or Cochran-Armitage trend analysis. A two-tailed *p*-value of ≤0.05 was considered significant.

## Results

**Fig. 1:**
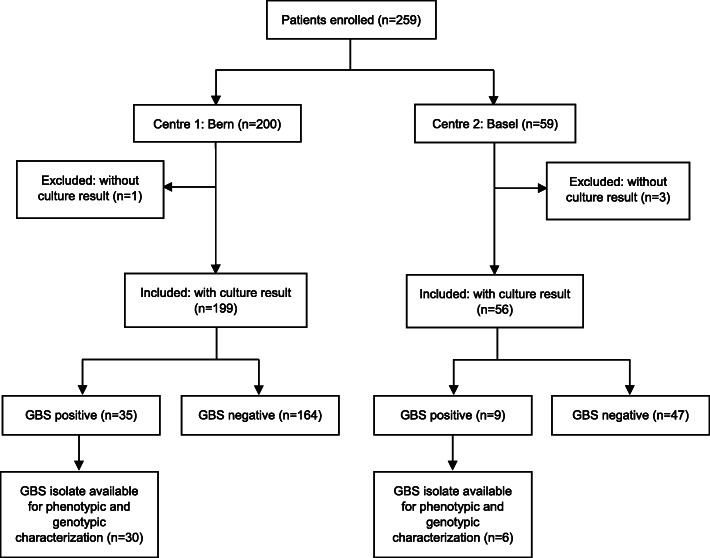
GBS Cite study flowchart

During the study period, 263 samples were collected from a total of 259 patients from the two centres, as shown in Fig. 1. Four samples were excluded from analysis, as they were obtained from the same patients during a second clinic visit. Another four were excluded because the samples could not be cultured. Thus, a total of 255 unique samples were included in the study.

Overall, the GBS positivity rate was 17% (44/255 positive samples), which was similar in both study sites (Bern centre: 18%, 35/199; Basel centre: 16%, 9/56). The results of the questionnaire data, including participants’ demographic characteristics and risk factors for GBS colonization, were analysed overall and divided by GBS carrier status, a summary of which is shown in Table 1. No significant associations were found between the participants’ demographic characteristics, medical history, menstrual history, sexual activity and GBS status.

**Table 1:** Participants’ demographic characteristics and risk factors for GBS acquisition overall and by GBS carrier status

Participants’ characteristics/risk factors	Overall (***n*** = 255)	GBS positive (***n*** = 44)	GBS negative (***n*** = 211)	***P***-Value
**Demographic characteristics**				
Mean age at enrolment (years, SD)	68 (6)	68 (5)	69 (6)	0.59
Minimum-maximum age (years)	69–98	60–84	60–98	Na
Participants’ origin				0.46*
Swiss (%)	205/239 (86%)	38/42 (90%)	167/197 (85%)	
Other (%)	34/239 (14%)	4/42 (10%)	30/197 (15%)	
Not answered (%)	16/255 (6%)	2/44 (5%)	14/255 (7%)	*Na*
**Medical history**				
No. of participants with diabetes (%)	20/254 (8%)	3/44 (7%)	17/210 (8%)	> 0.99*
No. of participants with liver disease (%)	7/253 (3%)	0/44 (0%)	7/209 (3%)	0.60*
No. of participants with history of stroke (%)	6/253 (2%)	1/44 (2%)	5/209 (2%)	> 0.99*
No. of participants with bladder weakness (%)	84/246 (34%)	10/43 (23%)	74/203 (36%)	0.09
No. of participants with history of cancer (%)	43/251 (17%)	9/44 (20%)	34/207 (16%)	0.51
No. of participants still receiving cancer treatment (%)	17/39 (44%)	4/9 (44%)	13/30 (43%)	> 0.99*
**Menstruation**				
Mean age of first menstruation (years, SD)	14 (2)	14 (2)	14 (2)	0.97
Not answered	14/255	1/44	13/211	Na
Mean age of menopause (years, SD)	49 (7)	48 (8)	49 (7)	0.69
Not answered	25/255	2/44	23/211	Na
**Sexual activity**				
No. of sexual partners during life				0.73^§^/0.80°
1 or less (%)	77/247 (31%)	13/42 (31%)	64/205 (31%)	0.97
2 or 3 (%)	80/247 (32%)	14/42 (33%)	66/205 (32%)	0.88
3 or 4 (%)	42/247 (17%)	5/42 (12%)	37/205 (18%)	0.33
5 or more (%)	48/247 (20%)	10/42 (24%)	38/205 (19%)	0.43
Not answered (%)	8/255 (3%)	2/44 (4%)	6/211 (3%)	Na
No. of participants with a new sexual partner in the last few months before enrolment	11/255 (4%)	1/44 (2%)	10/211 (5%)	0.69*
No. of participants’ sexual encounters in the 6 months before enrolment				0.98^§^*/0.65°
1 or less (%)	141/235 (60%)	24/41 (59%)	117/194 (60%)	0.83
2 or 3 (%)	15/235 (6%)	2/41 (4%)	13/194 (7%)	> 0.99*
3 or 4 (%)	18/235 (8%)	3/41 (7%)	15/194 (8%)	> 0.99*
At least once per month (%)	31/235 (13%)	6/41 (15%)	25/194 (13%)	0.76
At least once per week (%)	30/235 (13%)	6/41 (15%)	24/194 (12%)	0.69
Not answered (%)	20/255 (8%)	3/44 (7%)	17/211 (8%)	Na

Association between age variables and GBS status was investigated by using the Mann-Whitney test; association between other risk factors and GBS status was assessed by using the chi-square test or Fisher’s exact test (indicated by *); distribution analysis was also performed by using the chi-square test (indicated by §), Fisher’s exact test (*) or Cochran-Armitage trend analysis (°). In each analysis, the denominator includes only participants who provided the information; participants who did not answer the question were excluded. *SD* Standard deviation, *Na* Not applicable

Of the 44 swabs that tested positive, 36 GBS isolates were available for phenotypic and genotypic characterization (Fig. 1). The results are presented in Table 2. All 36 GBS isolates were susceptible to penicillin, with an MIC ranging between 0.032 and 0.094 mg/L. Eight GBS isolates (22%) were not susceptible to erythromycin, and three of them (3/8, 37.5%) had a MIC of ≥256 mg/L. Five isolates (14%) were not susceptible to clindamycin, four of them (4/5, 80%) with a MIC of ≥256 mg/L and one with a MIC of 1.5 mg/L. In addition, three GBS isolates (8%) that were considered clindamycin susceptible by E-test (MIC 0.19 mg/L) showed the MLS_B_ phenotype when tested by the double disk diffusion test, indicating inducible clindamycin resistance. Hence, eight (22%) GBS isolates were considered non-susceptible to clindamycin. Twenty-seven GBS isolates (75%) were susceptible to both clindamycin and erythromycin.

**Table 2:** Phenotypic and genotypic characterization results of GBS isolates

	No. of isolates (%)
**Drug susceptibility testing**	
Penicillin susceptible	36/36 (100%)
Clindamycin susceptible^a^	28/36 (78%)
Clindamycin non-susceptible^b^	8/36 (22%)
Erythromycin susceptible	28/36 (78%)
Erythromycin non-susceptible	8/36 (22%)
MLS_B_ phenotype (inducible clindamycin resistance)	3/36 (8%)
L phenotype (clindamycin resistant, erythromycin susceptible)	1/36 (3%)
M phenotype (clindamycin susceptible, erythromycin resistant)	1/36 (3%)
Clindamycin + Erythromycin non-susceptible^a^	4/36 (11%)
Clindamycin + Erythromycin non-susceptible^b^	7/36 (19%)
**Serotyping**	
Serotype III	12/36 (33%)
Serotype V	11/36 (31%)
Serotype Ia	6/36 (17%)
Serotype Ib	2/36 (5%)
Serotype II	2/36 (5%)
Serotype IV	2/36 (5%)
Serotype VI	1/36 (3%)
**Multilocus sequence typing**	
ST - no exact match	5/36 (14%)
ST1	4/36 (11%)
ST19	4/36 (11%)
ST175	3/36 (8%)
ST23	2/36 (6%)
ST249	2/36 (6%)
ST297	2/36 (6%)
Other STs	14/36 (38%)
**Phenotype association with genotype**	
Clindamycin + Erythromycin susceptible/non-susceptible^a^	***p*** **= 0.0129**
ST1	0/2
Non-ST1	27/2
Clindamycin + Erythromycin susceptible/non-susceptible^b^	***p*** **= 0.0008**
ST1	0/4
Non-ST1	27/3
MLS_B_ phenotype negative/positive	***p*** **= 0.0060**
ST1	0/2
Non-ST1	29/1

*MLS*_*B*_ Macrolide-lincosamide-streptogramin A, *ST* Sequence type as determined by multilocus sequence typing

^a^Excluding isolates showing inducible clindamycin resistance by double disk diffusion test

^b^Including isolates showing inducible clindamycin resistance

One GBS isolate belonging to capsular serotype Ia and ST624 (3%) showed an M phenotype, with erythromycin resistance only (MIC 4 mg/L). One GBS isolate assigned to capsular serotype III and ST19 (3%) displayed the L phenotype, being resistant to clindamycin only (MIC 1.5 mg/L).

Capsular serotyping showed that the most prevalent serotypes were III (33%, 12/36), V (31%, 11/36) and Ia (17%, 6/36). Multilocus sequence typing analysis showed that the most common sequence types were ST1 and ST19, accounting for four isolates each (11%); ST175 with three isolates (8%); and ST23, ST249 and ST297 with two isolates each (6%). Five strains could not be assigned to a sequence type (no exact match, 14%), while the remaining 14 isolates belonged to unique sequence types, including one to ST17 and capsular serotype III.

Compared with non-ST1 isolates, ST1 (serotype V) isolates were significantly associated with resistance to both clindamycin and erythromycin (*p* = 0.0129, when considering constitutive resistance only; *p* = 0.0008, when also including inducible clindamycin resistance) and to the MLS_B_ phenotype (*p* = 0.0060, Table 2). This association was based on small absolute numbers (*n* < 5).

## Discussion

The number of invasive GBS infections in the elderly population is continuously increasing [5], but the reason for this phenomenon is unclear. In Denmark, between 2005 and 2018 the incidence of invasive GBS in adults above the age of 65 years old increased more than twofold (from 3.23 to 8.34 per 100,000). Similar trends have been reported in other countries [8–12]. The prevalence of comorbidities that increase in parallel with age or an increase in GBS colonization have been discussed as potential contributing factors. Although the prevalence of GBS colonization in pregnant women has been investigated in numerous studies, with an average estimate around 20%, the prevalence in the elderly population – notably a group with increasing invasive GBS infections – is unknown. The GBS colonization rate is associated with sexual experience and activity [22, 23]. Considering that sexual activity in older people can change over time [24] and may have increased in recent decades, we aimed to determine the vaginal GBS colonization rate in elderly women. Our study showed a prevalence of GBS colonization of 17% in postmenopausal women (mean age, 68 years), similar to that reported by Moltó-Garcia et al. (17.8%) in Spain [25]. Edwards et al. reported a colonization rate of 21.7% in 254 healthy elderly participants (mean age, 73 years) in 2005 [15]. Kaplan et al. found a prevalence of GBS colonization of 12% among 167 elderly home residents (median age, 84 years) in 1983 [26]. These data indicate that the GBS colonization rate in pregnant women and healthy elderly adults is similar [16].

All GBS isolates preserved susceptibility to penicillin. However, compared with our previous study conducted on GBS isolates from pregnant women tested between 2009 and 2010 in the same geographical area, we observed a higher proportion of isolates that were non-susceptible to erythromycin (22% vs 14.6%), to clindamycin (14% vs 8.2%), and to both clindamycin and erythromycin (11% vs 7.7%) and that displayed inducible clindamycin resistance (8% vs 5.8%) [20]. However, a scientific comparison is not possible, because no longitudinal data were obtained. Moltó-Garcia et al. reported a similar resistance rate to erythromycin (23.4%) among their GBS samples collected between 2011 and 2012. Although they detected a higher prevalence of constitutive clindamycin resistance (20.6%), they observed the MLS_B_ phenotype in only 0.9% of their strains. Increasing trends of resistance were also reported elsewhere [7–11, 25, 27]. It is possible that elderly individuals were exposed to antibiotics more frequently than were pregnant women, and hence, GBS isolates display a higher resistance rate. However, we did not make such a comparison in our study.

We found one GBS isolate with L phenotype (clindamycin resistant but erythromycin susceptible). This phenotype is rare and may occur via the inactivation of lincosamide-specific nucleotidyl-transferases encoded by *lnu* genes [28]. Alternatively, this unusual mechanism of resistance may also be mediated by the ABC transporter, encoded by *lsaC* genes, and be responsible for cross-resistance to streptogramin A (LSA phenotype) and pleuromutilins (LSAP phenotype) [29, 30]. Recently, there have been increasing reports of such a phenotype in the United States, Europe, China, Korea and other countries, with a prevalence ranging from 0.26% in Italy to 15.9% in Korea [31–34]. This phenotype was detected in one of the most common clones circulating worldwide, ST19. This observation is worrisome because clindamycin is a frequently used alternative in patients with documented allergy to penicillin.

The most prevalent capsular serotypes in our population were III, V and Ia [20]. ST1, ST19 and ST23 were the predominant clones, accounting for 28% of our isolates. All three STs have been consistently reported to be significantly associated with asymptomatic colonization because of their limited invasive ability [14]. However, when it belongs to capsular serotype V, ST1 has been related to invasive disease, and a possible origin from a bovine ancestor has been hypothesized, similar to the case for hypervirulent clone ST17 [35]. Likewise, ST23 was found in carriage and invasive isolates [36]. Clone ST17 was identified in only one strain.

We confirmed significantly higher rates of resistance to macrolides and clindamycin associated with the genetic background of ST1, belonging to clonal complex 1, as previously described [27]. However, in contrast to Lopes et al., who reported the association of ST1 and capsular serotype Ib, we observed a relation to capsular serotype V [27]. The association of ST1 and capsular serotype V has also been described elsewhere [37].

Our study has limitations. Because of slow recruitment, the study was terminated prior to reaching the calculated target sample size, ending in a relatively small study population. However, the number of participants was comparable to (or even larger) than those in previous studies, and the GBS prevalence was similar to that of a study that included 600 individuals [15, 25, 26]. We only obtained vaginal and not recto-vaginal swabs, and may have missed an unknown proportion of GBS colonized individuals. However, we are convinced that the potential difference between the two sampling methods did not influence significantly the overall GBS colonization rate in our study population. Eight GBS isolates were lost for phenotypic and genotypic analysis (Fig. 1). Given the lack of association between risk factors, resistance testing and serotype, it is unlikely that the results of these eight lost GBS isolates would have changed the overall findings.

## Conclusions

The GBS vaginal colonization rate in women aged 60 or more was 17%. The observed increase in invasive GBS infections in elderly women may be for reasons other than the colonization rate. We found no associations with patient characteristics, comorbidities, menstrual history, menopause or sexual activity. Twenty-two percent of the isolates were not susceptible to clindamycin, and this pattern was associated with ST1. The most frequently found capsular serotypes were III and V. Our results indicate that the prevalence of colonization, the antibiotic susceptibility and the molecular patterns are similar in pregnant and elderly women.

## Supplementary Information

**Additional file 1.**

## Data Availability

The data sets used and/or analysed during the current study may be made available upon reasonable written request to the corresponding author.

## Abbreviations

GBS: Group B Streptococcus
ST: Sequence type
MIC: Minimum inhibitory concentration
MLS_B_: Macrolide-lincosamide-streptogramin B
