# Supplementary material for: Group B streptococcal colonization in elderly women
Source: BMC Infect Dis. 2021 May 3;21:408. doi: 10.1186/s12879-021-06102-x (PMC8091692; doi:10.1186/s12879-021-06102-x)
Supplement: Supplementary file 1 — Additional file 1. [file 12879_2021_6102_MOESM1_ESM.docx]

**Recruitment centre: No Name, ONLY CODE: ____________________**

**1. Origin / Ethnicity – irrespective of citizenship / passport**

You are __________________________________

For example: „Swiss“ or „Japanese“ or „Iranian“ or „South African“ etc.

**2. Health**

2.1. Are you diabetic?

□ No □ Yes

2.2. Do you have a liver disease or does a disease affect your liver?

(for example viral disease, alcohol disease, cancer)

□ No □ Yes

2.3. Did you experience a „Stroke“ previously?

(„cerebral ischemia“ or „cerebral haemorrhage“)

□ No □ Yes

2.4. Do you or did you have a bladder weakness (e.g., urinary incontinence)?

□ No □ Yes

2.5. Do you or did you have a cancer?

□ No □ Yes → Are you still receiving treatment? □ No □ Yes

↓

since when treatment was stopped?_____ (year)

**3. Menstruation**

3.1. How old were you when you experienced your first menstruation?____________

3.2. Dou you still have your menstruations? □ Yes □ No

3.3 How old were you when you stopped having menstruations? ________________

**4. Sexual activity**

4.1. How many sexual partners did you have in your life?

□ ≤1 □ 2 – 3 □ 3 – 4 □ ≥5

4.2. In the last few months, did you have a NEW sexual partner?

□ Yes □ No

4.3 In the last 6 months, how often did you have sex?

□ ≤1 □ 2 – 3 □ 3 – 4 □ ≥1x per month □ ≥1x per week
